# Regulation Involved in Colonization of Intercellular Spaces of Host Plants in *Ralstonia solanacearum*

**DOI:** 10.3389/fpls.2017.00967

**Published:** 2017-06-08

**Authors:** Yasufumi Hikichi, Yuka Mori, Shiho Ishikawa, Kazusa Hayashi, Kouhei Ohnishi, Akinori Kiba, Kenji Kai

**Affiliations:** ^1^Laboratory of Plant Pathology and Biotechnology, Kochi UniversityKochi, Japan; ^2^Research Institute of Molecular Genetics, Kochi UniversityKochi, Japan; ^3^Graduate School of Life and Environmental Sciences, Osaka Prefecture UniversityOsaka, Japan

**Keywords:** *Ralstonia solanacearum*, colonization of intercellular spaces, quorum sensing, methyl 3-hydroxymyristate, methyl 3-hydroxypalmitate, ralfuranones, virulence

## Abstract

A soil-borne bacterium *Ralstonia solanacearum* invading plant roots first colonizes the intercellular spaces of the root, and eventually enters xylem vessels, where it replicates at high levels leading to wilting symptoms. After invasion into intercellular spaces, *R. solanacearum* strain OE1-1 attaches to host cells and expression of the *hrp* genes encoding components of the type III secretion system (T3SS). OE1-1 then constructs T3SS and secrets effectors into host cells, inducing expression of the host gene encoding phosphatidic acid phosphatase. This leads to suppressing plant innate immunity. Then, OE1-1 grows on host cells, inducing quorum sensing (QS). The QS contributes to regulation of OE1-1 colonization of intercellular spaces including mushroom-type biofilm formation on host cells, leading to its virulence. *R. solanacearum* strains AW1 and K60 produce methyl 3-hydroxypalmitate (3-OH PAME) as a QS signal. The methyltransferase PhcB synthesizes 3-OH PAME. When 3-OH PAME reaches a threshold level, it increases the ability of the histidine kinase PhcS to phosphorylate the response regulator PhcR. This results in elevated levels of functional PhcA, the global virulence regulator. On the other hand, strains OE1-1 and GMI1000 produce methyl 3-hydroxymyristate (3-OH MAME) as a QS signal. Among *R. solanacearum* strains, the deduced PhcB and PhcS amino acid sequences are related to the production of QS signals. *R. solanacearum* produces aryl-furanone secondary metabolites, ralfuranones, which are extracellularly secreted and required for its virulence, dependent on the QS. Interestingly, ralfuranones affect the QS feedback loop. Taken together, integrated signaling *via* ralfuranones influences the QS, contributing to pathogen virulence.

## Introduction

**FIGURE 1:**
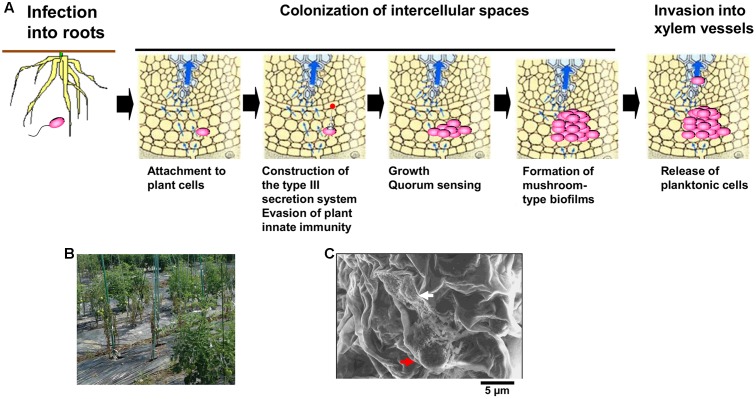
Infection route of *Ralstonia solanacearum* in intercellular spaces of roots **(A)**, bacterial wilt of tomato plants caused by *R. solanacearum* in a field located in Kochi university, Japan **(B)**, and microcolony (white-colored arrow) and the mushroom type-biofilm formed by cells of *R. solanacearum* strain OE1-1 (red-colored arrow) on tomato cells observed by the scanning electron microscope **(C)**.

A soil-borne plant pathogenic bacterium *Ralstonia solanacearum* normally invades plant roots through wounds or natural openings. The pathogen first colonizes the intercellular spaces of the root, and eventually enters xylem vessels and spreads up into stems through the xylem (**Figure 1A**; Hikichi, 2016). Reduced sap flow caused by the presence of many bacterial cells and exopolysaccharide (EPS) slime produced by the bacteria in some xylem vessels leads to wilting symptoms (**Figure 1B**; Genin and Denny, 2012). Molecular traits such as EPS production in *R. solanacearum* infecting xylem vessels have been thus focused in its virulence mechanisms. On the contrary, colonization of intercellular spaces is required for *R. solanacearum* virulence (Hikichi, 2016), molecular traits regulating its colonization of intercellular spaces has remained unclear. In this mini review, we focus molecular traits regulating colonization of intercellular spaces of host plants in *R. solanacearum*, especially integrated intracellular/intercellular signaling with the quorum sensing (*phc* QS) involved in its virulence.

## *R. solanacearum* Strain OE1-1 Produces Mushroom-Type Biofilms

After invading intercellular spaces, *R. solanacearum* strain OE1-1 first attaches to host cells and produces microcolonies following by mBFs (**Figures 1A,C**; Mori et al., 2016). The mBF formation is essential for colonization of intercellular spaces by OE1-1, leading to its virulence.

## Regulation of *hrp* Genes

**FIGURE 2:**
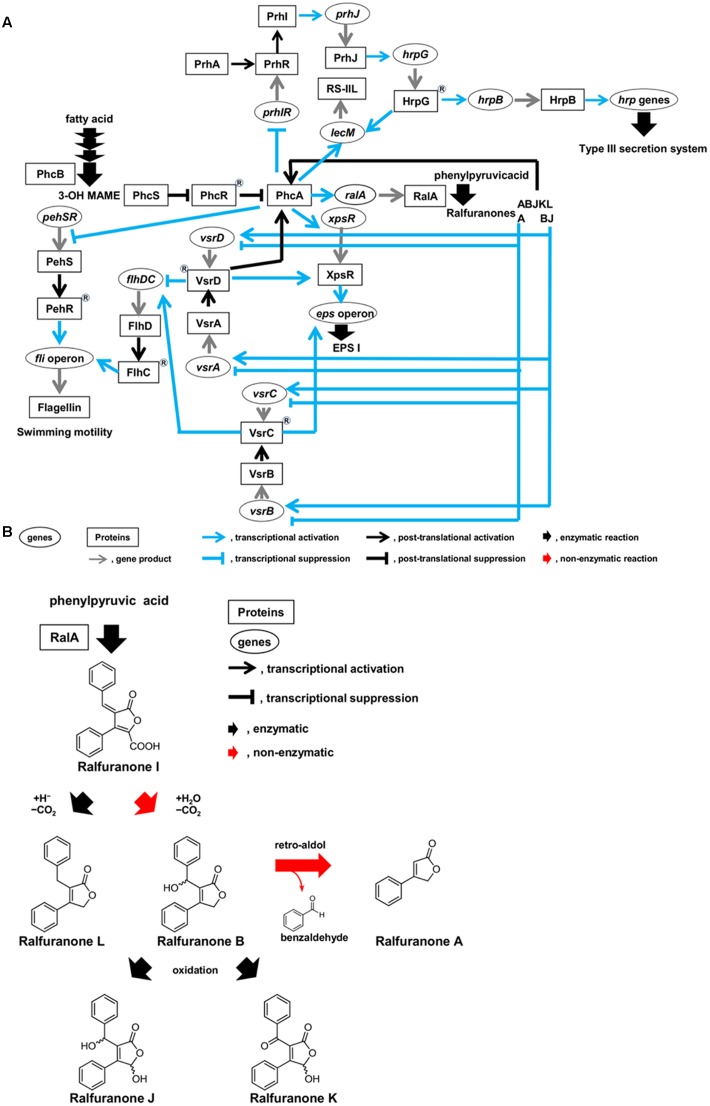
Circuits of molecular traits involved in colonization of intercellular spaces **(A)** and schematic diagram of synthesis route of ralfuranones **(B)** in *R. solanacearum* strain OE1-1 during activation of quorum sensing. *R. solanacearum* strain OE1-1 produces methyl 3-hydroxymyristate (3-OH MAME) as a quorum sensing signal by PhcB, a putative methyltransferase, and ralfuranone I as a precursor of the other ralfuranones by RalA, a tridomain non-ribosomal peptide synthetase-like furanone synthetase. Ralfuranone I is non-enzymatically converted into ralfuranone B in the supernatant. The non-enzymatic elimination of benzaldehyde from ralfuranone B produces ralfuranone A. Ralfuranones J and K are the products of enzymatic oxidation of ralfuranone B. Ralfuranone L is enzymatically synthesized from ralfuranone I.

*Ralstonia solanacearum* has *hrp* genes encoding structural constituents of the type III secretion system (T3SS), which translocates effectors into host cells (Genin and Denny, 2012). This activation is sensed by the outer membrane receptor PrhA, which transduces signals through the PrhI and PrhR anti-sigma-sigma system and a complex regulatory cascade integrated by PrhJ, HrpG, and HrpB regulators (**Figure 2A**). A MarR family transcriptional regulator, PrhN, is also involved in positive regulation of T3SS (Zhang et al., 2015).

After invading intercellular spaces, OE1-1 induces expression of *lecM* encoding a lectin RS-IIL by HrpG, leading to its attachment into host cells (**Figure 2A**; Mori et al., 2016). It is thus thought that OE1-1 synchronizes attachment into host cells with T3SS construction.

## Evasion of Plant Innate Immunity by *R. solanacearum* Invading Intercellular Spaces of Host Plants

In tobacco plants, the invasion of intercellular spaces by *R. solanacearum* induces Sec14P-mediated phospholipid signaling which produces phosphatidic acid (PA) in chloroplast membranes (Kiba et al., 2012, 2014). The PA is involved in induction of the defense system mediated by jasmonic acid and reactive oxygen species. The expression of the gene encoding phosphatidic acid phosphatase (PAP) is induced in tobacco plants by invasion of the virulent *R. solanacearum* strain OE1-1, but not the avirulent strain 8107 (Nakano et al., 2013). The PAP dephosphorylates PA into diacylglycerol, interfering with the induction of jasmonic acid- and reactive oxygen-mediated plant innate immunity. The translocation of effectors into tobacco cells through the T3SS leads to decreased levels of PA, interfering with the induction of PA-mediated plant innate immunity and allowing OE1-1 to grow on tobacco cells (**Figure 1A**; Hikichi, 2016).

## The Quorum Sensing of *R. solanacearum*

The expression of pathogenicity factors in *R. solanacearum* is controlled by a complex regulatory network that responds to environmental conditions, the presence of host cells, and bacterial density (Schell, 2000; Genin and Denny, 2012). At the center of this network is a LysR family transcriptional regulator, PhcA (**Figure 2A**; Brumbley et al., 1993), which coordinates the expression of several virulence factors including the major ESP, EPS I (Huang and Schell, 1995). The function of PhcA is regulated in response to cell density by the *phc* QS (Flavier et al., 1997).

After evading the innate immunity of host plants, *R. solanacearum* vigorously grows on host cells, inducing the *phc* QS (**Figure 1A**; Mori et al., 2016). The levels of functional PhcA via the *phc* QS are controlled by the *phcBRS* operon (Genin and Denny, 2012). The *R. solanacearum* strains AW1 and K60 produce methyl 3-hydroxypalmitate (3-OH PAME) as a QS signal (Flavier et al., 1997; Kai et al., 2015). Additionally, *R. solanacearum* strain OE1-1 produces methyl 3-hydroxymyristate (3-OH MAME) as a QS signal (Kai et al., 2015). These QS signals are synthesized by PhcB, a putative methyltransferase (Flavier et al., 1997; Kai et al., 2015). When the QS signals reach a threshold level, they induce the ability of the histidine kinase PhcS to phosphorylate the response regulator PhcR (**Figure 2A**; Schell, 2000; Genin and Denny, 2012; Papenfort and Bassler, 2016). It has been thought that the phosphorylation of PhcR reduced its binding activity to PhcA, resulting in elevated levels of functional PhcA. Therefore, cells of *R. solanacearum* at higher densities (>10^6^ cfu/ml) have abundant functional PhcA and produce multiple virulence factors such as EPS I while suppressing the production of survival and invasion factors such as T3SS and swimming motility (Genin and Denny, 2012; Hikichi, 2016).

Phylogenetic trees constructed using amino acid sequences of PhcB and PhcS, but not PhcR, cluster 18 strains of *R. solanacearum* into two groups according to their QS signal types; 3-OH MAME or 3-OH PAME (Kai et al., 2015). The types of *phc* QS signals do not reflect the locations from which they were isolated, the phylotypes, or the host plants from which the strains were isolated, the races. Thus, the ancestors of *R. solanacearum* might have first coevolved the QS signal synthase (PhcB) and its receptor (PhcS), and then evolved the QS-dependent signaling for the adaptation to new and different environments.

Interestingly, functional PhcA suppresses expression of the *prhIR* operon, leading to the suppression of *hrp* gene expression (**Figure 2A**; Genin et al., 2005; Yoshimochi et al., 2009). The *phc* QS-deficient mutants lose their ability to colonize intercellular spaces and cannot invade xylem vessels and lose virulence (Mori et al., 2016), similar to *hrp* mutants (Hikichi, 2016). Furthermore, expression of *lecM* is also induced by functional PhcA through the *phc* QS (Meng et al., 2015; Mori et al., 2016). The *lecM*, of which expression is induced by functional PhcA, is involved in control development of mBFs, suggesting that the *phc* QS controls mBF formation by strain OE1-1 (Mori et al., 2016). Therefore, the *phc* QS dependent PhcA-mediated regulation allows *R. solanacearum* to control the elaborate and tunable regulation of its colonization of intercellular spaces, leading to its virulence.

## Feedback Regulation of the *phc* QS by Ralfuranones

*Ralstonia solanacearum* synthesizes aryl-furanone secondary metabolites known as ralfuranones A, B, I, J, K, and L, which are extracellularly secreted (**Figure 2B**; Pauly et al., 2013; Kai et al., 2014). Ralfuranone I is a precursor of the other ralfuranones. The production of transaminase and furanone synthase, which are encoded by *ralD* and *ralA*, respectively, depends on functional PhcA *via* the *phc* QS system. Both the transaminase and furanone synthase are involved in the biosynthesis of ralfuranone I (Schneider et al., 2009; Wackler et al., 2011; Kai et al., 2014). Ralfuranone I is non-enzymatically converted into ralfuranone B in the supernatant (Kai et al., 2016). The non-enzymatic elimination of benzaldehyde from ralfuranone B produces ralfuranone A, while ralfuranones J and K are the products of enzymatic oxidation of ralfuranone B. Ralfuranone L is enzymatically synthesized from ralfuranone I. Thus, ralfuranone production is dependent on the *phc* QS system.

Ralfuranone productivity is involved in the full virulence of *R. solanacearum* strain OE1-1 (Kai et al., 2014). A ralfuranone-deficient mutant (Δ*ralA*) exhibits significantly less EPS I production and significantly enhanced the swimming motility than strain OE1-1 (Mori et al., 2017). Quantitative real-time PCR assays reveal that Δ*ralA* expresses *phcB* and *phcA* at levels similar to those in strain OE1-1. In contrast, *R. solanacearum* transcriptome data generated by RNA sequencing technology shows that Δ*ralA* exhibits downregulated expression of more than 90% of QS-positively regulated genes including EPS I production-related genes, type VI secretion system-related genes, plant cell wall degradation enzyme genes (*pme*, *egl*, and *pehC*), acyl-homoserine lactones-two component system-related genes (*solI* and *solR*) and some effector genes secreted through T3SS (*ripG4*, *ripG5*, *ripO1*, *ripTP5*, *ripS*, and *ripAU*) (Mori et al., 2017). Furthermore, Δ*ralA* exhibits upregulated expression of more than 75% of QS-negatively regulated genes including flagellar motility-related genes, T3SS-related genes, some effector genes secreted through T3SS (*ripAX1, ripA2, ripAF1, ripX, ripAB, ripAC, ripS2, ripS3, ripQ*, *ripAZ1*, and *ripAD*), and chemotaxis-related genes.

Ralfuranone supplementation restores EPS I production-related *epsB* expression, which is induced by the QS in strain OE1-1, in Δ*ralA*, restoring its ability to aggregate dependently on EPS I production (Mori et al., 2017). Additionally, expression of flagellar motility-related *fliC* expression, which is negatively regulated by the *phc* QS, is suppressed in Δ*ralA* by application of ralfuranones A and B, restoring its swimming motility to wild-type levels.

Together, post-translational regulation through ralfuranones may affect the QS feedback loop.

## Regulation of the Two-Component Systems, VsrAd and VsrBc, By Ralfuranones

The PhcA and VsrAD two-component sensor/response regulatory systems are necessary for full activation of *xpsR* transcription (**Figure 2A**; Genin and Denny, 2012). Additionally, both the transcriptional regulator XpsR and the response regulator VsrC upregulate the expression of the *eps* operon (Huang et al., 1998; Garg et al., 2000). EPS I production is thus influenced by the VsrAD and VsrBC two-component systems. These two-component systems also regulate flagella biogenesis (Genin and Denny, 2012). Interestingly, ralfuranone A is involved in the negative regulation of *vsrAD* and *vsrBC* expression (Mori et al., 2017). In contrast, ralfuranones B and J positively regulate the expression of *vsrA*, *vsrD*, *vsrB*, and *vsrC*. Therefore, the integrated regulation of *vsrAD* and *vsrBC* expression by ralfuranones A, B, and J contributes to EPS I production and swimming motility. Furthermore, VsrAD is upstream of PhcA and is involved in the biosynthesis of ralfuranones (Schneider et al., 2009). Therefore, the expression of *vsrAD* may be feedback-regulated through ralfuranones A, B, and J, leading to the regulation of PhcA function.

## Conclusion

*Ralstonia solanacearum* invading intercellular spaces of roots attaches to plant cells and constructs the T3SS, translocating effectors into plant cells through the T3SS and evading induction of plant innate immunity. *R. solanacearum* then grows on plant cells and activates the *phc* QS. The *phc* QS contributes to control of mBF formation by *R. solanacearum* on plant cells, leading to its colonization of intercellular spaces required for its virulence. *R. solanacearum* produces ralfuranones A, B, I, J, K, and L dependently on the *phc* QS, and the extracellular secretion of each ralfuranone by OE1-1 changes over time. During the early stages of infection, 3-OH MAME-mediated intercellular signaling activates the *phc* QS, leading to the production and secretion of ralfuranones. Each ralfuranone then mediates intercellular signaling between *R. solanacearum* cells in association with the feedback loop of the *phc* QS. The integrated intracellular/intercellular signaling of OE1-1 cells *via* each ralfuranone coupled with *phc* QS elaborately and tunably regulate molecular traits during colonization of intercellular spaces by *R. solanacearum* strain OE1-1, leading to its virulence (**Figure 2A**).

## Author Contributions

YM, SI, and KH performed experiments and analyzed data. KO designed the research, analyzed data, and wrote the manuscript. AK designed the research. KK and YH designed the research, performed experiments, analyzed data, and wrote the manuscript.

## Conflict of Interest Statement

The authors declare that the research was conducted in the absence of any commercial or financial relationships that could be construed as a potential conflict of interest.
